# Engineered baker’s yeast as whole-cell biocatalyst for one-pot stereo-selective conversion of amines to alcohols

**DOI:** 10.1186/s12934-014-0118-z

**Published:** 2014-08-12

**Authors:** Nora Weber, Marie Gorwa-Grauslund, Magnus Carlquist

**Affiliations:** Division of Applied Microbiology, Department of Chemistry, Lund University, Lund, SE-22100 Sweden

**Keywords:** Transamination, Reduction, VAMT, Saccharomyces cerevisiae, Capsicum chinense, Lactobacillus kefir, NADPH regeneration, Pyruvate, PLP, Kinetic resolution, Asymmetric synthesis

## Abstract

**Background:**

One-pot multi-step biocatalysis is advantageous over step-by-step synthesis as it reduces the number of process operation units, leading to significant process intensification. Whole-cell biocatalysis with metabolically active cells is especially valuable since all enzymes can be co-expressed in the cell whose metabolism can be exploited for supply of co-substrates and co-factors.

**Results:**

In this study, a heterologous enzymatic system consisting of ω-transaminase and ketone reductase was introduced in *Saccharomyces cerevisiae*, and evaluated for one-pot stereo-selective conversion of amines to alcohols. The system was applied for simultaneous kinetic resolution of *racemic* 1-phenylethylamine to (*R*)-1-phenylethylamine and reduction of the ketone intermediate to (*R*)-1-phenylethanol. Glucose was used as sole co-substrate for both the supply of amine acceptor and the regeneration of NADPH in the reduction step.

**Conclusions:**

The whole-cell biocatalyst was shown to sustain transaminase-reductase-catalyzed enantioselective conversion of amines to alcohols with glucose as co-substrate. The transamination catalyzed by recombinant vanillin aminotransferase from *Capsicum chinense* proved to be the rate-limiting step as a three-fold increase in transaminase gene copy number led to a two-fold increased conversion. The (*R*)-selective NADPH-dependent alcohol dehydrogenase from *Lactobacillus kefir* proved to be efficient in catalyzing the reduction of the acetophenone generated in the transamination reaction.

## Background

Chiral amine and/or chiral alcohol moieties are present in many bioactive molecules, e.g. Saxagliptin, which is used for the treatment of type 2 diabetes; Vanlev, an antihypertensive drug; Rivastigmine for the treatment of Alzheimer’s disease and the HIV protease inhibitors Atazanavir and Indinavirsulphate [1–3]. Consequently, efficient methods for preparation of bioactive compounds with chiral amine and/or alcohol functionalities, including their inter-conversion and inversion of stereochemistry are needed. Chemical methods where chiral amines are first transformed into intermediates and subsequently into chiral alcohols with inversion of the stereochemistry have been previously described [4–6]. Additionally, a mixture of diethylene glycol and KOH at 210°C converts several amines into alcohols in a one-pot reaction, albeit with limited enantiomeric purity [7], which highlights main limitations of chemical synthesis. As an alternative, biocatalytic methods based on isolated alcohol oxidase and ω-transaminase enzymes for one-pot conversion of alcohols to amines [8] and conversion of amines to alcohols with ω-transaminase-oxidoreductase enzymes [9] have been described. However, these methods rely on the addition of multiple enzymes, expensive co-substrates and *in vitro* co-factor regeneration systems. As an alternative, whole-cell biocatalysis with recombinant *Pichia pastoris* over-expressing a transaminase combined with endogenous reductase activity has previously been described for the conversion of amines to alcohols [10]. Additionally, it has been demonstrated, that recombinant *Saccharomyces cerevisiae* expressing ω-transaminase can be used for kinetic resolution of *racemic* amines with glucose as sole co-substrate for the supply of amine acceptor and pyridoxal-5’-phosphate by cell metabolism [11]. Engineered yeast has also been developed for efficient stereo-selective carbonyl reductions of prochiral ketones for the synthesis of chiral alcohols (see review [12] or [13]). In that case, cell metabolism was exploited for *in vivo* regeneration of the NADPH co-factor [14,15].

**Figure 1:**
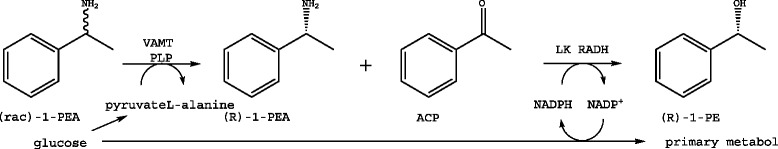
**Reaction scheme for simultaneous production of (** ***R*** **)-1-phenylethylamine (PEA) and (** ***R*** **)-1-phenylethanol (PE).**

Herein, we study the use of recombinant yeast as whole-cell biocatalyst for one-pot enantioselective conversion of amines to chiral alcohols, using the kinetic resolution of *racemic* 1-phenylethylamine (PEA) to (*R*)-1-PEA and simultaneous production of (*R*)-1-phenylethanol (1-PE) as a model reaction (Figure 1). Enantiomerically pure (*R*)-1-PEA and (*R*)-1-PE are for instance important building blocks for the production of 4-amino-6-aryl thienopyrimidines and Fendiline derivatives, which can be used for the treatment of cancer, as well as for *N*^3^-substituted uridines with CNS-depressant effect or (*S*)-Rivastigmine for the treatment of Alzheimer’s disease [16–19]. In the present study, the possibility to co-express a functional heterologous ω-transaminase ketone reductase (TA-KRED) gene cascade, and to exploit the inherent cellular machinery to generate amine acceptors and for regeneration of NADPH via sugar metabolism has been studied. The influence of a three-fold increase of the transaminase gene copy number was evaluated as well as the effect of resting or growing cells on conversion. The specific reaction cascade does not exist in wild type yeast, and the influence on cell physiology of the catalytic activity of the artificially assembled enzymes has not been described yet.

## Results

### Transamination and product inhibition

**Figure 2:**
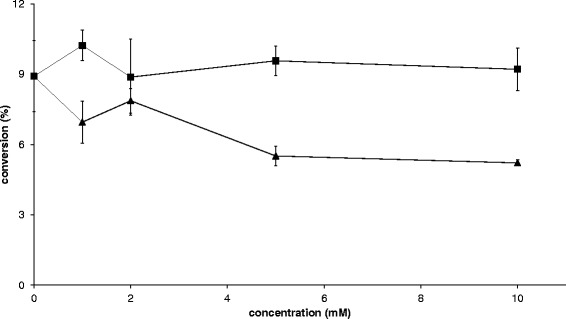
**Kinetic resolution of** ***racemic*** **1-PEA at varying (co-) product concentration after 24 h with VAMT strain (TMB4350).** (square) = L-alanine, (triangle) = acetophenone (ACP).

Whole-cell transamination was previously achieved in engineered *S. cerevisiae* strains carrying a codon-optimised vanillin aminotransferase (*VAMT*) gene from *Capsicum chinense* [11]. However, the reaction did not go to completion. A decrease of the transaminase reaction rate due to substrate and/or (co-) product inhibition has previously been observed [20–22]. So we investigated the effect of different concentrations of L-alanine and acetophenone (ACP) on the efficiency of the kinetic resolution of (*rac*)-1-PEA by strain TMB4350 (VAMT). The transamination reaction with whole-cells was followed with concentrations of L-alanine and ACP ranging from 0 to 10 mM at the start of the reaction. The addition of L-alanine did not lead to any negative effect on the kinetic resolution of (*rac*)-1-PEA (Figure 2). In contrast, already 1 mM ACP inhibited kinetic resolution of (*rac*)-1-PEA (Figure 2) and the conversion decreased to about half when 10 mM ACP were added. It was therefore decided to further reduce ACP to a chiral alcohol, which would limit the inhibition of the transamination reaction, while generating another valuable chiral alcohol product.

### Assembly of functional transaminase - oxidoreductase cascade in yeast

Codon-optimized genes encoding *VAMT* from *Capsicum chinense* [23] and the (*R*)-specific alcohol dehydrogenase (*LK RADH*) from *Lactobacillus kefir* [24] were assembled in series in a chromosomal integration cassette generating plasmid pNW4 (Table 1). The integrative plasmid was used to transform the strain TMB4150, generating VAMT + LK RADH strain (TMB4351). A plasmid containing only *VAMT* gene and no reductase was also constructed (pNW10, see Table 1), and used to transform the same strain TMB4150, generating strain TMB4367 (VAMT). Both strains contained identical copy numbers of the integrated plasmid, which was verified by qPCR (data not shown).

**Table 1:** **Strains and plasmids used in this study**

**Plasmids and strains**	**Description**	**Reference**
pUC57 VAMT	Codon-optimised *VAMT* encoding gene; *AMP* resistance gene	[11]
pUC57 LK RADH	Codon-optimised *LK RADH* encoding gene; *AMP* resistance gene	GenScript, NJ, USA
YIplac128-HXT7’p-PGKt	Plasmid with HXT7 truncated promoter and PGK terminator; *AMP* resistance gene; *LEU2* gene	[15]
YIpOB7	TDH3 promoter, ADH1 terminator, *XDH* under PGK1 promoter, with PGK1 terminator; *URA3* gene; *AMP* resistance gene	[25]
pNW2	*VAMT* gene under HXT7 truncated promoter, with PGK1 terminator, *LEU2* gene; *AMP* resistance gene	[11]
pNW4	*VAMT* under TDH3 promoter, with ADH1 terminator; *LK RADH* under PGK1 promoter, with PGK1 terminator; *URA3* gene; *AMP* resistance gene	This study
pNW10	*VAMT* under TDH3 promoter, with ADH1 terminator; *URA3* gene; *AMP* resistance gene	This study
*S. cerevisiae* TMB4150	*CEN.PK2-1C MAT*a *ura3-52 MAL2-8* ^*C*^ *SUC2, TRP1, LEU2, HIS3*	Jan Knudsen, unpublished
*S. cerevisiae* TMB4350	CEN.PK113-16B containing pNW2, overexpressing *VAMT* encoding gene	[11]
*S. cerevisiae* TMB4351	TMB4150 containing pNW4, overexpressing *VAMT* and *LK RADH* encoding genes, *TRP1*, *LEU2*, *HIS3*	This study
*S. cerevisiae* TMB4365	TMB4150 containing 2 times more copies of pNW2 than TMB4350, overexpressing *VAMT* encoding gene, *TRP1*, *HIS3*	This study
*S. cerevisiae* TMB4367	TMB4150 containing pNW10, overexpressing *VAMT* encoding gene, *TRP1*, *LEU2*, *HIS3*	This study
*S. cerevisiae* TMB4373	TMB4365 pNW4, overexpressing *VAMT* and *LK RADH* encoding genes, *TRP1*, *HIS3*	This study

Strains VAMT (TMB4367) and VAMT + LK RADH (TMB4351) were evaluated in a batch conversion of (*rac*)-1-PEA to (*R*)-1-PEA and (*R*)-1-PE. In particular, the specific activity, enantioselectivity and the ability to exploit the native sugar metabolism to provide essential co-factors and co-substrates for the reaction were determined.

**Figure 3:**
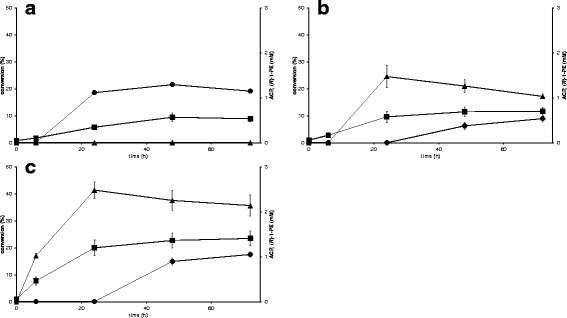
**Kinetic resolution of** ***racemic*** **1-PEA with (a) VAMT strain (TMB4367), (b) VAMT + LK RADH strain (TMB4351) and (c) 3x VAMT + LK RADH strain (TMB4373).** (square) = conversion (%), (circle) = ACP (mM), (triangle) = (*R*)-1-PE.

For the VAMT strain, about 1.1 mM ACP was formed after 24 h of reaction, and thereafter it remained at this concentration range until 72 h (Figure 3a). The ACP reductase activity in cell extract was low (0.0099 ± 0.0075 U/mg for NADH and 0.0057 ± 0.0008 U/mg for NADPH), which is in accordance with what has been observed in previous studies [11]. Thus, as expected there was no formation of 1-phenylethanol from acetophenone from yeast endogenous reductase activity. Overexpression of the NADPH dependent reductase from *Lactobacillus kefir* in VAMT + LK RADH strain led to 45 times higher activity with NADPH as co-factor (0.2598 ± 0.0302 U/mg) and about 2 times higher activity with NADH (0.017 ± 0.0088 U/mg) in cell extract. And during the first 24 h of the whole-cell conversion of *racemic* 1-PEA, there was no ACP formation, but a significant production of enantiomerically pure (*R*)-1-PE (>99% *ee*) instead (Figure 3b). In later stages of the reaction, (*R*)-1-PE decreased from 1.48 mM at 24 h to 1.03 mM at 72 h while ACP accumulation was observed (0.54 mM at 72 h).

During the first 24 h, the reaction rate of (*S*)-1-PEA to ACP with VAMT + LK RADH strain (TMB4351) was with 0.014 mmol (*S*)-1-PEA/g dw/h nearly two-fold higher than for VAMT strain (TMB4367) with 0.008 mmol (*S*)-1-PEA/g dw/h (see Figure 3a and b). It decreased between 24 h and 48 h and stopped after that for both strains at a substrate conversion of about 12% (VAMT + LK RADH) and 9% (VAMT), respectively. Altogether, the kinetic resolution of (*rac*)-1-PEA was improved by 25% for VAMT + LK RADH strain (TMB4351) compared to VAMT strain (TMB4367).

### Effect of increasing the amount of gene copy numbers of transaminase

To investigate if the limitation of the two-step reaction lied in the transamination step, a strain with elevated transaminase activity and unchanged reductase activity as compared to VAMT + LK RADH strain (TMB4351) was constructed by increasing *VAMT* gene copy number. Strain TMB4365 containing several copies of *VAMT* gene was transformed with the plasmid pNW4 containing VAMT and LK RADH generating strain TMB4373 which contained three times more copies of *VAMT* gene than VAMT + LK RADH strain (TMB4351). The increased gene copy number of *VAMT* led to a two-fold higher conversion for 3x VAMT + LK RADH strain (TMB4373) (Figure 3c) compared to VAMT + LK RADH strain (TMB4351) containing only one copy of *VAMT* and *LK RADH* genes (Figure 3b) and nearly three-fold higher conversion than for VAMT strain (TMB4367) (Figure 3a). The higher amount of transaminase increased the overall reaction rate of the kinetic resolution from 0.014 mmol (*S*)-1-PEA/g dw/h (TMB4351, 1x VAMT + 1x LK RADH) to 0.018 mmol (*S*)-1-PEA/g dw/h (TMB4373, 3x VAMT + 1x LK RADH) during the first 24 h. However, even with three copies of *VAMT* gene present, the reaction rate decreased during the reaction (between 48 h and 72 h, no detectable rate with VAMT + LK RADH strain (TMB4351) and 0.001 mmol (*S*)-1-PEA/g dw/h with 3x VAMT + LK RADH strain (TMB4373). As with VAMT + LK RADH strain (TMB4351), 3x VAMT + 1x LK RADH strain (TMB4373) showed no ACP production during the first 24 h, indicating that the reductase was efficient in reducing ACP to (*R*)-1-PE (Figure 3c). Between 24 h and 72 h however, nearly twice the amount of ACP (1.05 mM) was formed with 3x VAMT + LK RADH strain (TMB4373) than with VAMT + LK RADH strain (0.54 mM, TMB4351). For both strains, the highest amount of (*R*)-1-PE (>99% *ee*) was at 24 h, after which it was decreasing at about the same rate (0.002 mmol (*R*)-1-PE/g dw/h for VAMT + LK RADH strain, 0.001 mmol (*R*)-1-PE/g dw/h for 3x VAMT + LK RADH strain).

### Resting vs. growing cells as biocatalyst

**Figure 4:**
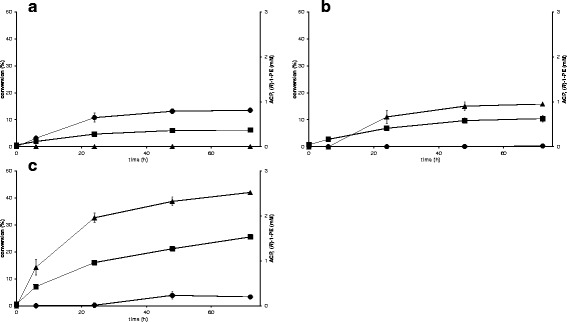
**Kinetic resolution of (** ***rac*** **)-1-PEA with growing cells of (a) VAMT strain (TMB4367), (b) VAMT + LK RADH strain (TMB4351) and (c) 3x VAMT + LK RADH strain (TMB4373).** (square) = conversion (%), (circle) = ACP (mM), (triangle) = (*R*)-1-PE.

Many biotransformations with whole-cells are carried out with resting cells as no biomass is produced simultaneously which leads to high specific activities and yields from the added energy source [26]. However, resting cells can lose their activity faster than growing cells due to a shortage of intracellular NADPH and no possibility for synthesis of new enzymes, due to limited nutrient resources [26]. Therefore, the effect of using growing instead of resting cells as whole-cell biocatalyst was investigated by performing the reaction in defined mineral media with VAMT strain (TMB4367, Figure 4a), VAMT + LK RADH strain (TMB4351, Figure 4b) and 3x VAMT + LK RADH strain (TMB4373, Figure 4c). The conversion was for all three strains very similar between resting and growing cells. Only during 48 h to 72 h for the 3x VAMT + LK RADH strain (TMB4373), the use of growing cells led to a 8% higher conversion. A sample taken after 140 h showed though, that the reaction did not continue further (data not shown) with the growing cells.

There was no significant difference in the product formation for resting and growing cells of VAMT strain (TMB4367, Figure 4a). On the contrary for VAMT + LK RADH strain (TMB4351), resting cells accumulated 0.54 mM ACP after 72 h (Figure 3b) but only 0.04 mM with growing cells (Figure 4b). After 72 h, the amount of (*R*)-1-PE was about 1 mM with both resting and growing cells, but for resting cells, nearly 1.5 mM of (*R*)-1-PE was produced after 24 h.

3x VAMT + LK RADH strain (TMB4373) showed five times higher production of ACP with resting (1 mM, see Figure 3c) than with growing cells (0.2 mM, see Figure 4c). The highest amount of (*R*)-1-PE (2.5 mM) was obtained with resting cells also after 24 h and 2.1 mM (*R*)-1-PE was left after 72 h; for growing cells 2.5 mM (*R*)-1-PE was formed after 72 h.

### Glucose assimilation during the reaction

**Figure 5:**
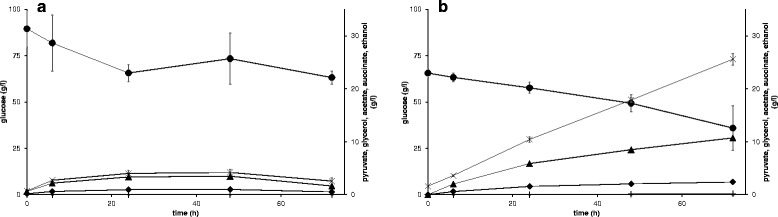
**By-product formation from glucose during kinetic resolution with TMB4373 (VAMT (three copies) + LK RADH).** (circle) = glucose, (horizontal line) = pyruvate, (vertical line) = succinate, (triangle) = glycerol, (diamond) = acetate, (cross) = ethanol. **(a)** resting and **(b)** growing cells.

To assess the metabolic activity of the cells during one-pot biocatalysis, glucose and by-product formation was monitored during the kinetic resolution of (*rac*)-1-PEA with resting and growing cells of the best biocatalyst (3x VAMT + LK RADH strain, TMB4373). With resting cells, the glucose consumption rate was 1.08 mmol glucose/g dw/h for the first 24 h and reached then a plateau which led to an arrest in NADPH co-factor regeneration (see Figure 5a). Growing cells, on the other hand, had nearly a constant glucose consumption rate of 0.44 mmol glucose/g dw/h until 72 h (Figure 5b). For both strains, no pyruvate and nearly no succinate were formed, as both are intermediate compounds and generally not observed outside the cells during fermentation of glucose [27]. There was though a significant difference with the other by-products, about five times more acetate, seven times more glycerol and ten times more ethanol were formed with growing cells compared to resting ones, reflecting increased metabolic activity.

## Discussion

In this study, we demonstrate that recombinant yeast over-expressing heterologous transaminase and reductase genes can be used as whole-cell biocatalyst for one-pot conversion of specific amine enantiomers to alcohols with high selectivity.

The biocatalytic system only contains yeast, buffer, the *racemic* substrate phenylethylamine (PEA), glucose as co-substrate and pyridoxal-5’-phosphate (PLP) as co-factor, which makes it environmentally friendly, simple and cheap as compared to chemical reaction systems that often require work-up of intermediates, expensive catalysts, protective groups or several solvents. The process is also much simpler than *in vitro* enzymatic counterpart. For instance, simultaneous synthesis of (*R*)-1-PEA and (*R*)-1-PE with purified enzymes consisted of the following steps: Growth of *E. coli* cells containing ω-TA and GDH (glucose dehydrogenase), harvest, disruption, dialysis of the cell extract (addition of PLP for ω-TA) and commercially obtained ADH (alcohol dehydrogenase), addition of purified enzymes, NADPH, (*rac*)-1-PEA, pyruvate, glucose and buffer [9]. Multi-step reactions are especially suitable with whole-cell systems since all enzymes necessary for operational synthetic cascade reactions can be co-expressed in the same host and thus have a minimal number of upstream steps, which results in simplified processes. For example, several steps for expression, purification, or addition of involved enzymes, co-factors, co-substrates or intermediates can be omitted which significantly reduce the cost of the process [28].

Baker’s yeast *S. cerevisiae* is used in many different industrial settings such as production of ethanol, butanol, isobutanol, succhinic acid, artemisinin and recombinant protein production [29]. Additionally, research is carried out for the production of phenolic compounds like flavonoids and stilbenoids [29]. It is also a well suited biocatalyst for the enantioselective conversion of (*S*)-1-PEA to (*R*)-1-PE as there is no competing endogenous transaminase activity [11] and very little ACP reductase activity. In addition, glucose can fuel both catalytic reactions: it is converted to the central carbon intermediate pyruvate that acts as the amine acceptor in the transamination reaction and it also regenerates, via the pentose phosphate pathway, the co-factor NADPH needed for the carbonyl reduction. Thus, there is no need for external addition of neither of these system functions, which simplifies and reduces the cost of the process [30].

Coupling of the transamination to a subsequent reduction of the product also led to a 25% improvement of the kinetic resolution of the *racemic* amine substrate, which was probably due to the lower level of ACP inhibitor. In parallel, incomplete inhibition by ACP might be due to the use of whole-cells instead of isolated enzyme, as the transaminase was not directly exposed to the bulk concentration of ACP. Previous comparison of whole-cells and cell extract of *Pichia pastoris* showed that the ω-transaminase was indeed inhibited stronger by ACP and at much lower concentration in cell extract than in whole-cells [10].

L-Alanine was found not to inhibit the transamination reaction in the whole-cell system. This is consistent with previous studies using purified ω-transaminases from *Vibrio fluvialis* JS17, *Bacillus thuringiensis* JS64 and *Klebsiella pneumonia* JS2F where addition of the co-product L-alanine did not inhibit the reaction to a high extent at low concentrations [22,31]. The inhibitory effect in our set-up was even lower. This may, again, be explained by the use of a whole-cell system instead of enzymes. Also, in *S. cerevisiae*, L-alanine can be converted by *ALT1* gene product to pyruvate, converting α-ketoglutarate into L-glutamate, [32], or by *AGX1* gene product to pyruvate, yielding L-glycine from glyoxylate [33]. The whole-cell system is therefore beneficial for kinetic resolution where L-alanine is a by-product, as it can be removed from the reaction, shifting the thermodynamic equilibrium to the product side.

A higher amount of gene copies of the transaminase *VAMT* led to significant improvement in biocatalysis, indicating that the transamination reaction was the bottleneck of the cascade system. The reaction rate of the following NADPH-dependent reductase from *L. kefir* was, at least during the first 24 h, as fast as the reaction rate of the transaminase, as no ACP was built up. After 24 h however, ACP started accumulating, maybe because of the reduced rate of NADPH co-factor regeneration (see below).

Biocatalytic systems based on whole-cells can have major differences in the metabolic state of the cell. Resting and growing cells are both alive and metabolically active, but the addition of vitamins, trace elements and a nitrogen source increase the cost of the process. Additionally, nucleic and amino acids, lipids and other compounds are synthesized, the growing cells are less robust and cellular reactions compete for co-factors [34]. There was no significant difference between growing and resting cells for the transamination step in our system, which indicates that the higher glycolytic flux did not lead to more available pyruvate. Alternatively, pyruvate from glucose assimilation was not limiting the reaction in any of the process set-ups. Therefore, a buffer system would be preferred as it is simpler and cheaper than a system with media as the reaction solution. However, a significant difference was observed between resting and growing cells for the reduction step, thereby confirming previous observations on diketone whole-cell reduction [14]. With growing cells, the amount of ACP was 14 times lower for VAMT + LK RADH strain (TMB4351) and five times lower for 3x VAMT + LK RADH strain (TMB4373) than with resting cells. Low accumulation of ACP shows that the transamination and reduction rates are similar with growing cells, even after 24 h of reaction. This was more pronounced when only one copy of VAMT was present, as ACP was generated less rapidly by the cells. Additionally, after 72 h the amount of (*R*)-1-PE was 20% higher with growing than with resting cells with 3x VAMT + LK RADH strain (TMB4373). A higher amount of ACP can have two reasons: (1) The transamination is more efficient, and/or (2) the reduction is less efficient. Growing cells displayed higher glucose consumption rate and product formation from fermentation of glucose [34,35]; however, the intracellular concentration of pyruvate was not measured and its influence on the difference in reaction performance is therefore not known. A comparison between using glucose or adding pyruvate directly as co-substrate for whole-cell transamination with resting cells has been performed previously [11]. The use of glucose as co-substrate had a higher initial specific reaction rate, but led to about the same final conversion as when pyruvate was added as co-substrate. This indicates that pyruvate is not limiting the reaction for resting cells. The higher glycolytic flux for growing cells could lead to faster regeneration of the co-factor NADPH [34] and therefore more efficient reduction of ACP. These observations lead to the conclusion that for resting cells, the amount of NADPH was too low after 24 h. When glucose consumption after 24 h decreased, less NADPH may have been regenerated and NADP^+^ may have accumulated, leading to a shift of the reduction towards the ketone side, which would explain the depletion of (*R*)-1-PE with resting cells after 24 h. The accumulation of ACP is due to further kinetic resolution of *racemic* 1-PEA after 24 h. For a transaminase-oxidoreductase coupled system, growing cells are therefore more suitable, unless more efficient NADPH regeneration systems can be introduced. For this strategy *PGI1* gene is deleted and *ZWF1* gene overexpressed, which forces the glycolytic flux through the pentose phosphate pathway and leads to efficient regeneration of NADPH for whole-cell reduction [15].

## Conclusions

We could demonstrate simultaneous kinetic resolution of *racemic*-1-PEA to (*R*)-1-PEA and reduction of the ketone intermediate ACP to (*R*)-1-PE. Glucose was used as sole co-substrate for production of the amine acceptor pyruvate and regeneration of the co-factor NADPH. Our method shows the possibility of one-pot two-step reactions with whole-cell systems. Even if the method cannot yet compete in an industrial setting, it shows the potential of microbial cell factories for the production of useful chiral building blocks.

## Methods

### Chemicals

All chemicals were bought from VWR (Leuven, Belgium) except acetophenone (ACP), *racemic* 1-phenylethylamine (1-PEA), (*R*)-1-PEA, (*S*)-1-PEA and sodium pyruvate from Merck (Hohenbrunn, Germany), (*R*)-1-phenylethanol (PE) and (*S*)-1-PE from Sigma-Aldrich (Steinheim, Germany) and pyridoxal-5’-phosphate (PLP) from AppliChem (Darmstadt, Germany).

### Strains, media, and cell growth

*S. cerevisiae* strain TMB4150 (*MAT*a, *ura3-52 MAL2-8*^*C*^*SUC2*) was kindly provided by Jan Knudsen, Applied Microbiology, Lund University, Sweden. *E. coli* strain DH5α (Life Technologies, Rockville, MD, USA) was used for subcloning. *S. cerevisiae* strain TMB4350 [11] was used for the inhibition experiments. TMB4351, TMB4367 and TMB4373 (see construction below and Table 1) were used for whole-cell transamination and measurement of reductase activity. Strains were kept as 20% glycerol stocks at −80°C and grown on solid media for 1–2 days prior to experiments. Transformation and cell growth was performed as described previously [11] except that mineral medium [36] was used instead of YPG medium.

### Nucleic acid manipulation

Plasmid DNA was prepared with the GeneJET Plasmid Miniprep Kit (Thermo Scientific, Rockford, IL, USA) and agarose gel DNA extraction was performed using QIAquick® Gel Extraction Kit (Qiagen GmbH, Hilden, Germany). Primers from MWG-Biotech AG (Ebersberg, Germany) and *Phusion* Hot Start II DNA Polymerase and dNTPs from Thermo Scientific (Rockford, IL, USA) were used for polymerase chain reactions (PCR) and performed in a GeneAmp PCR system 9700 (Applied Biosystems, Foster City, CA, USA). PCR products were purified with the GeneJET PCR Purification Kit (Thermo Scientific, Rockford, IL, USA). Sequencing was performed by MWG-Biotech AG (Ebersberg, Germany). InFusion® HD Cloning Kit (Clontech Laboratories, Mountain View, CA, USA) was used for DNA manipulation.

### Strain construction

Plasmids pUC57 VAMT containing the ω-transaminase gene from *Capsicum chinense* [GenBank: AAC78480.1, Swiss-Prot: O82521] and pUC57 LK RADH containing the NADPH-dependent reductase from *Lactobacillus kefir* [GenBank: AAP94029.1, Swiss-Prot: Q6WVP7] (Table 1) were PCR amplified with the primers listed in Table 2. YIpOB7 was digested with the restriction enzyme *Xba*I (VAMT) or *Bgl*II (LK RADH) and the PCR fragments inserted by InFusion® cloning, thus creating pNW4 and pNW10. Correct orientation of the inserts and sequences were verified by restriction analysis and sequencing. Integrative vectors pNW4 and pNW10 were cleaved with *Apa*I within the *URA3* marker gene and then used to transform the haploid laboratory strain TMB4150 (*MAT***a**, *ura3-52 MAL2-8*^*C*^*SUC2*) which resulted in strains TMB4351 and TMB4367.

**Table 2:** **Primers used in this study**

**Name**	**Sequence 5’ → 3’**
VAMT fwd	TTCGACGGATTCTAGATGGCAAACATTACAAACGAATTCA
VAMT rev	AGTCCAAAGCTCTAGTTATTGCTTTTGGGACTTCAATTCTTC
LK RADH fwd	TAAAACCAAAAGATCATGACTGACAGATT
LK RADH rev	GAGACATGGGAGATCTTATTGAGCAGTGT
VAMT qPCR fwd	CAGATTGGCAAACAACTTGG
VAMT qPCR rev	CGAAATATGTGGCTGGAGG

TMB4373 was constructed by transformation of TMB4365 with pNW4.

### Determination of relative gene copy number

Strains were grown in 5 ml YPG medium overnight, total DNA was extracted with Yeast DNA Extraction Kit (Thermo Scientific, Rockford, IL, USA) and the concentration measured with BioDrop μLite (BioDrop, Cambridge, UK). The gene copy number of the strains was determined by qPCR with a LightCycler® Nano Instrument (Roche Applied Science, IN, USA) with the following program: 95°C, 60 sec; 45x (95°C, 10 sec; 55°C, 20 sec; 72°C, 30 sec); 40°C, 60 sec. The mastermix contained 1 U Ex Taq HS polymerase (TaKaRa Bio Inc, Otsu, Shiga, Japan), 1x Ex Taq buffer, 0.2 mM dNTPs, 0.5 μM primers (see Table 2) and 1x EvaGreen (Biotium, Hayward, CA, USA). A duplicate standard curve was performed with TMB4367 (VAMT) as reference and 0.05, 0.1, 1.0, 10.0 and 20.0 ng total DNA. Duplicates of 0.1 and 10 ng total DNA of the other strains were compared to the standard curve and the resulting value was coupled to the relative amount of gene copies. The formation of the correct fragment was verified by a melting curve analysis (50°C to 97°C at 0.05°C/sec) and an agarose gel.

### Whole-cell transamination

50 ml sealed serum flasks with magnetic stirring (140 rpm) and 5 g/l cell dry weight (dw) at 30°C were used for whole-cell transamination. For resting cells, the reaction solution contained 10 ml 100 mM sodium phosphate buffer (pH 7.0), 50–100 g/l glucose, 12–25 mM (*rac*)-1-PEA and 0.1 mM PLP. Vitamins, trace elements, (NH_4_)_2_SO_4_, MgSO_4_ and ergosterol were also added as in the growth media [36] for growing cells. For the inhibition experiment, 0–10 mM L-alanine or ACP were added from the start.

### Enzyme activity

For the determination of specific enzyme activity, cells were grown until stationary phase, washed once with 25 ml water, and resuspended in sodium phosphate buffer (100 mM, pH 7.0). Cell extract was made with glass beads (0.5 mm) and precellys 24 bead beater with a cryolys cooling unit (Bertin technologies, Aix-en-Provence Cedex, France). Total protein amount was determined by Bradford with bovine serum albumin (Fermentas UAB, Vilnius, Lithuania) as standard [37]. Reductase activity was assayed by following the oxidation of NAD (P) H at 340 nm using Ultrospec 2100pro spectrophotometer (Amersham Biosciences, Sweden). Data were collected using the software SWIFTII (Amersham Biosciences, Sweden). Samples were diluted so that the absorbance decreased linearly during 5 min. One unit of activity corresponds to 1 μmol NAD (P) H consumed per minute at 30°C. The assay contained sodium phosphate buffer (100 mM, pH 7.0), NAD (P) H (200 μM), ACP (10 mM) and cell extract.

### Analyses

Growth was monitored by measuring optical density at a wavelength of 620 nm (OD_620_) with an Ultrospec 2100pro spectrophotometer (Amersham Biosciences, Sweden). Cell dry weight determination was performed as described previously, as was determination of (*R*)-1-phenylethylamine, (*S*)-1-phenylethylamine, (*R*)-1-phenylethanol, (*S*)-1-phenylethanol, acetophenone, glucose, pyruvate, glycerol, acetate, succinate and ethanol [11].
